# Mutual inhibition of insulin signaling and PHLPP-1 determines cardioprotective efficiency of Akt in aged heart

**DOI:** 10.18632/aging.100933

**Published:** 2016-03-27

**Authors:** Yuan Xing, Wanqing Sun, Yishi Wang, Feng Gao, Heng Ma

**Affiliations:** ^1^ Department of Physiology, Fourth Military Medical University, Xi'an 710032, China; ^2^ Department of Cardiovascular Medicine, First Affiliated Hospital of Jilin University, Changchun 130000, China; ^3^ Department of Aerospace Medicine, Fourth Military Medical University, Xi'an 710032, China; ^4^ Department of Pathophysiology, Fourth Military Medical University, Xi'an 710032, China

**Keywords:** myocardial I/R, insulin, PHLPP-1, Akt, degradation

## Abstract

Insulin protects cardiomyocytes from myocardial ischemia/reperfusion (MI/R) injury through activating Akt. However, phosphatase PHLPP-1 (PH domain leucine-rich repeat protein phosphatase-1) dephosphorylates and inactivates Akt. The balanced competitive interaction of insulin and PHLPP-1 has not been directly examined. In this study, we have identified the effect of mutual inhibition of insulin signaling and PHLPP-1 on the cardioprotective efficiency of Akt in aged heart. Young (3 mon) and aged (20 mon) Sprague Dawley (SD) rats were subjected to MI/R *in vivo*. The PHLPP-1 level was higher in aged vs. young hearts at base. But, insulin treatment failed to decrease PHLPP-1 level during reperfusion in the aged hearts. Consequently, the cardioprotection of insulin-induced Akt activation was impaired in aged hearts, resulting in more susceptible to MI/R injury. In cultured rat ventricular myocytes, PHLPP-1 knockdown significantly enhanced insulin-induced Akt phosphorylation and reduced simulated hypoxia/reoxygenation-induced apoptosis. Contrary, PHLPP-1 overexpression terminated Akt phosphorylation and deteriorated myocytes apoptosis. Using *in vivo* aged animal models, we confirmed that cardiac PHLPP-1 knockdown or enhanced insulin sensitivity by exercise training dramatically increased insulin-induced Akt phosphorylation. Specifically, MI/R-induced cardiomyocyte apoptosis and infarct size were decreased and cardiac function was increased. More importantly, we found that insulin regulated the degradation of PHLPP-1 and insulin treatment could enhance the binding between PHLPP-1 and β-transducin repeat-containing protein (β-TrCP) to target for ubiquitin-dependent degradation. Altogether, we have identified a new mechanism by which insulin suppresses PHLPP-1 to enhance Akt activation. But, aged heart possesses lower insulin effectiveness and fails to decrease PHLPP-1 during MI/R, which subsequently limited Akt activity and cardioprotection. PHLPP-1 could be a promising therapeutic interventional target for elderly ischemic heart disease patients.

## INTRODUCTION

Accurate regulation of the balance between protein phosphorylation and dephosphorylation is fundamental for cellular homeostasis. Dysregulation of this balance results in pathophysiological states, driving cellular injury or cell death [1]. Akt is an established survival signal in the heart. Aberrant activation of Akt has been associated with cardiovascular disease, diabetes and cardiac aging [2]. Phosphorylation of post-translation is required for Akt activity. Conversely, dephosphorylation of Akt inactivates this kinase and limits the Akt survival signal pathway [3]. Therefore, Akt specific regulators (positive or negative) determine dynamic change of Akt activity. Though protein phosphatase (PP)2A, calcineurin (PP2B) and phosphatase and tensin homolog (PTEN) have been shown to inhibit Akt activity through regulating its upstream signaling or dephosphorylating Akt at Thr308 and/or Ser473, they have poor substrate selectivity, eliciting de-phosphorylation of diverse target molecules [4-6].

Over the past decade, a more specific Akt-directed protein phosphatase PHLPP has been identified, which dephosphorylates Akt at Ser473 terminating Akt signaling [7]. There are two isoforms of PHLPP, PHLPP-1 and PHLPP-2, negatively regulate different kinds of Akt isoforms in diverse tissue respectively. Particularly, PHLPP-1 is clearly detected and specific dephosphorylates Akt Ser473 in the heart [8]. Meanwhile, PHLPP-1 knockout mouse hearts show increased phosphorylation of Akt [9] and decreased infarct size following MI/R [8]. These studies provide important evidence that PHLPP-1 negatively regulates Akt phosphorylation and activation, and that deletion or inhibition of PHLPP-1 improves cardiomyocyte survival [4, 8]. However, several questions remain unanswered: Is there any relevant inhibitory mechanism of PHLPP1 under basal conditions or during MI/R? If PHLPP-1 predominated in heart, is it possible that PHLPP-1 weakens cardioprotection of Akt and leads to ischemic intolerance as seen in aged hearts?

Aging is one of the most important risk factors for cardiovascular accidents and aged heart is more susceptible to I/R injury. However, the underlying mechanisms of this vulnerability have not been fully identified [10]. The decreased phosphorylation of signal proteins in aged myocardium can be the result of either impaired phosphorylation by protein kinases or enhanced dephosphorylation by protein phosphatases [11]. Our [12, 13] and related studies [14-17] have showed that insulin elicits cardioprotection against MI/R injury through activating PI3K/Akt signaling. Treatment with insulin during reperfusion activates Akt and reduces post-ischemic myocardial apoptotic death [18, 19]. Phosphorylation of Akt contributes significantly to the anti-apoptotic effect of insulin [20]. Therefore, we have reason to suppose that the presence of PHLPP-1 would impair Akt phosphorylation response to insulin during reperfusion, which may weaken the myocardial protective effect of insulin in aged heart resulting aging-related ischemic intolerance.

Notably, to date, the majority of the studies on PHLPP-1 mainly demonstrate that it negatively regulates Akt [9, 21, 22], with little information regarding the self-stability of PHLPP-1 in the heart. Given the importance of phosphorylation for Akt maximal activation, it remains to be established whether insulin leads to diminished PHLPP-1 protein level and consequently enhances Akt activation as well as cardiomyocyte survival. For that matter, we tested the hypothesis that insulin removing the break on Akt activation by downregulation of PHLPP-1 accentuates physiological Akt activation, which exerts salutary effects in protecting the heart against I/R stress.

The balanced competitive interaction of insulin signaling and PHLPP-1 has not been directly examined. Therefore, the aims of the present study were: (1) to determine whether PHLPP-1 may contribute to the desensitization of insulin-Akt responses and cardioprotection decline in I/R injured aging heart; (2) to examine whether insulin plays a downregulating role in PHLPP-1 protein level and then intensify cardiac Akt activation.

## RESULTS

### Senescence compromises cardioprotection of insulin against MI/R injury

**Figure 1:**
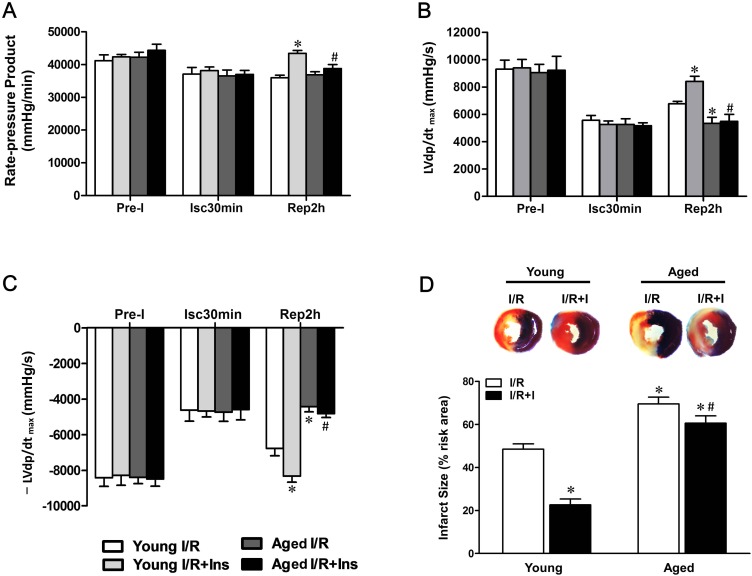
Impaired cardioprotection of insulin against MI/R injury in aged hearts Young and aged rats were subjected to 30 minutes of ischemia by LAD ligation and followed by 2 hours of reperfusion with or without insulin treatment (60 U/L, intravenous infusion at 4 mL/kg per h for 2 h, beginning 5 min before reperfusion). (**A**) Left ventricular develop products were calculated by rate-pressure product (RPP) [heart rate (beats per minutes) × ventricular pressure (mmHg)]. (**B** and **C**) Maximal rate of rise of left ventricular pressure (±LVdP/dt_max)_. (**D**) Representative sections of myocardial infarction and the percent of infarct size after 4 hours reperfusion (*P < 0.05 vs. Young I/R; ^#^*P* < 0.05 vs. Young I/R+Ins. Values are means ± S.E., n = 5 per group).

MI followed by reperfusion resulted in significant cardiac injury including myocardial infarction and cardiac contractile as well as diastolic dysfunction in young animals. But, when subjected to *in vivo* MI/R, aged animals manifested significantly greater cardiac injury than young controls (Fig.1). Specifically, cardiac function was further depressed evidencing by lower rate-pressure product (RPP) (Fig.1A) and ±LVdp/dt_max_ (Fig.1B, C), and MI/R-induced infarct size was enlarged (Fig.1D). Intriguingly, acute administration of insulin shortly before reperfusion (60 U/l, intravenous infusion at 4 mL/kg/h for 2 h, beginning at 5 min before reperfusion [23]) exerted cardioprotection against MI/R injury as evidenced by improved postischemic contractile function recovery (Fig.1A-C) and reduced infarct size in young hearts, whereas the cardioprotection of insulin was markedly decreased in aged hearts (Fig.1D). Administration of insulin exerted no significant protective effect on infarct size in aged hearts. These results indicated the ischemic vulnerability of aged hearts and insulin-induced cardioprotection against myocardial reperfusion injury is impaired in aged hearts versus young hearts.

### Sustained PHLPP-1 expression attenuates insulin-activated Akt phosphorylation in aged hearts

**Figure 2:**
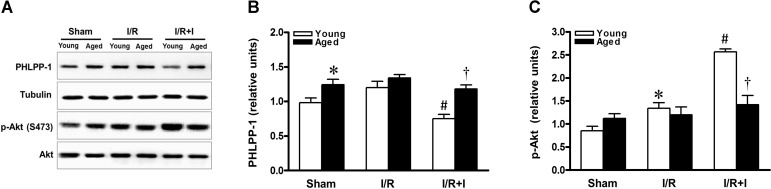
Sustained PHLPP-1 attenuated insulin-activated Akt phosphorylation Young and aged rats were subjected to sham, 30 minutes of *in vivo* ischemia and followed by 30 minutes of reperfusion with or without insulin treatment (60 U/L, intravenous infusion at 4 mL/kg per h for 30 minutes, beginning 5 min before reperfusion) and then hearts were collected for Western blots. (**A**) Representative immunoblots showing PHLPP-1 content and phosphorylation of Akt at Ser473 from heart extracts of young and aged rats. The bar graphs show the relative levels of PHLPP-1 (**B**) and phosphorylation of Akt at Ser473 (**C**), respectively (*P < 0.05 vs. Young sham; ^#^*P* < 0.05 vs. Young I/R; ^†^ *P* < 0.05 vs. Young I/R+Ins. Values are means ± S.E., n = 5 per group).

Previous study demonstrated that phosphorylation of Akt served as the central mediator for the cardio-protective effects of insulin during myocardial reperfusion [12, 16, 24]. As summarized in Figure 2, PHLPP-1 expression was clearly detected in the heart *in vivo*, and the cardiac PHLPP-1 level was not appreciably changed during MI/R (30 min / 30 min) (Fig.2A). However, in young hearts, administration of insulin during reperfusion remarkably decreased PHLPP-1 expression (Fig.2B). Consistent with PHLPP-1 downregulation, Akt phosphorylation was increased in response to MI/R and there was a significantly further enhancement of Akt Ser473 (the best-characterized PHLPP-1 substrate [7]) phosphorylation in insulin-treated young rats (Fig.2C). The PHLPP-1 level was higher in aged vs. young hearts at base (Fig.2A). Importantly, in the aged hearts, insulin treatment failed to decrease PHLPP-1 level during reperfusion, thus without enhancing phosphorylation of Akt at Ser473 (Fig.2C). These data evoked that the sustained presence of PHLPP-1 blocks insulin-induced enhancement of Akt phosphorylation during myocardial reperfusion in aged hearts.

### PHLPP-1 protein level significantly affects insulin-activated Akt

**Figure 3:**
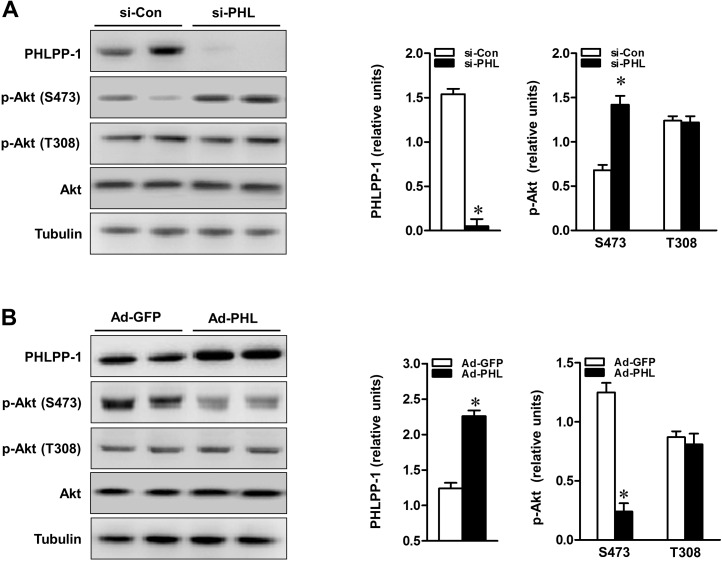
PHLPP-1 expression level affected Akt phosphorylation at Ser473 (**A**) Cardiomyocytes were transfected with PHLPP-1-specific (si-PHL) and scrambled negative control (si-Con) siRNA for 48 hours. Representative immunoblots demonstrating the two sites of Akt phosphorylation with PHLPP-1 knockdown (left panel). The bar graphs (right panel) show the relative levels of PHLPP-1 and Akt phosphorylation (S473 & T308, normalized by total Akt bands), respectively. (**B**) Cardiomyocytes were infected with the recombinant human-*phlpp-1* over-expression plasmid (Ad-PHL) and negative control (Ad-GFP) adenovirus for 72 hours. Representative immunoblots demonstrating the two sites of Akt phosphorylation with PHLPP-1 upregulation (left panel). The bar graphs (right panel) show the relative levels of PHLPP-1 and Akt phosphorylation (S473 & T308), respectively (*P < 0.05 vs. respective negative control. Values are means ± S.E., n = 5 per group).

To determine whether PHLPP-1 level regulates insulin-induced Akt phosphorylation, PHLPP-1 expression was up- and down-regulated in cardiomyocytes. Firstly, PHLPP-1 was also clearly detected in cultured neonatal rat ventricular myocytes (Fig.3A). Significant knockdown of PHLPP-1 protein levels was achieved by siRNA transfection of cardiomyocytes (Fig.3A).

**Figure 4:**
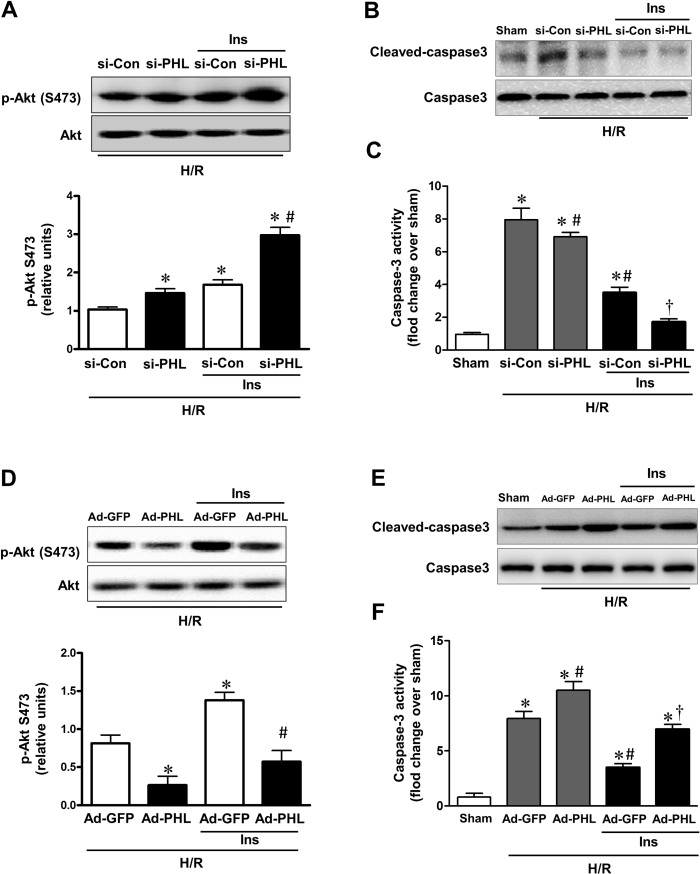
PHLPP-1 expression level affected insulin-activated Akt phosphorylation (**A**) Cardiomyocytes were transfected with PHLPP-1-specific (si-PHL) and scrambled negative control (si-Con) siRNA for 48 hours. Each group myocytes were subjected to hypoxia (3 h)/reoxygenation (3 h) with or without insulin treatment (100 nM). Representative immunoblots and bar graphs (right panel) show the relative levels of Akt phosphorylation (S473) (*P < 0.05 vs. Ad-sh-Con+H/R; ^#^*P* < 0.05 vs. Ad-sh-Con+H/R+Ins). (**B**) Immunoblotting of cleaved/total caspase 3. (**C**) Quantification of caspase-3 activity in H/R-induced cardiomyocytes of the indicated group (*P < 0.05 vs. sham; ^#^*P* < 0.05 vs. si-Con+H/R; ^†^*P* < 0.05 vs. si-Con+H/R+Ins). (**D**) Cardiomyocytes were infected with Ad-PHL or Ad-GFP for 72 hours. Each group myocytes were subjected to hypoxia (3 h)/reoxygenation (3 h) with or without insulin treatment (100 nM). Immunoblots showing the relative levels of Akt phosphorylation (S473) (*P < 0.05 vs. Ad-GFP+H/R; ^#^*P* < 0.05 vs. Ad-GFP+H/R+Ins). (**E**) Immunoblotting of cleaved/total caspase 3. (**F**) Quantification of caspase-3 activity in H/R-induced cardiomyocytes of the indicated group (*P < 0.05 vs. sham; ^#^*P* < 0.05 vs. Ad-GFP+H/R; ^†^*P* < 0.05 vs. Ad-GFP+H/R+Ins. Values are means ± S.E., n = 5 per group).

Phosphorylation of Akt at Ser473 was increased nearly two-folds in PHLPP-1 knockdown cardiomyocytes while phosphorylation of Thr 308 had no obvious change as shown previously [8, 9]. To evaluate the functional effect of PHLPP-1 knockdown, cardiomyo-cytes were subjected to hypoxia/reoxygenation (H/R) and levels of Akt phosphorylation were examined (Fig.4A). Knocking down of PHLPP-1 augmented Akt phosphorylation at Ser473 in response to H/R treatment. Notably, increased Akt phosphorylation with administration of insulin during reoxygenation was further enhanced by PHLPP-1 knockdown. To determine whether the increased Akt activity provided by PHLPP-1 knockdown translates into enhanced protection of cardiomyocytes, cleaved caspase-3 level and myocardial caspase-3 activity were determined. A robust apoptotic response, up-regulated cleaved caspase-3 level and caspase-3 activity which was induced by H/R, significantly decreased by PHLPP-1 knockdown, suggesting PHLPP-1 knockdown was protective (Fig.4B,C). Moreover, the insulin-mediated protection was greatly promoted in PHLPP-1 knockdown cardio-myocytes since the significantly increased Akt activity. These data provided evidence that insulin-mediated increase in phosphorylated Akt and anti-apoptotic effect were significantly potentiated by PHLPP-1 knockdown.

From another aspect, cardiomyocytes were infected with Adenoviruses packaging PHLPP-1 (Ad-PHL). Over-expression of PHLPP-1 decreased Akt phosphorylation at Ser473 (Fig.3B). Furthermore, Akt phosphorylation at Ser473 was significantly attenuated in response to H/R stress in PHLPP-1 over-expressed cardiomyocytes. We also found insulin-increased Akt phosphorylation was significantly blocked by PHLPP-1 over-expression (Fig.4D). In additionally, the protective effects of insulin treatment during reoxygenation were interdicted, as evidenced by increased cleaved caspase-3 level and caspase-3 activity (Fig.4E,F). Taken together, these data supported the notion that PHLPP-1 up-regulation restrains activation of Akt induced by insulin and suppresses myocytes survival.

### PHLPP-1 knockdown *in vivo* alleviated ischemic injury in aged hearts

**Figure 5:**
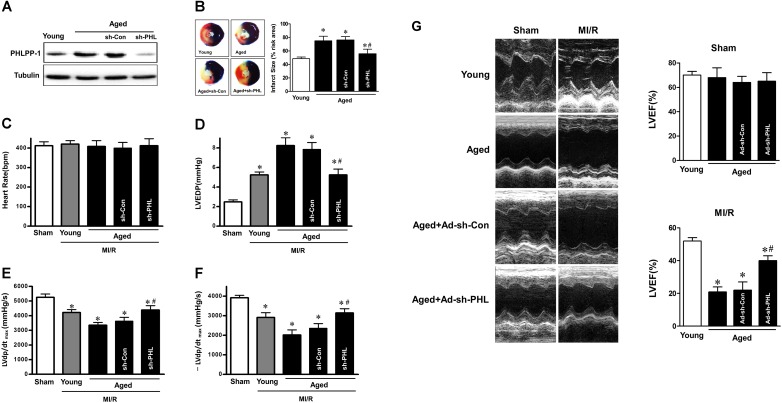
PHLPP-1 knockdown alleviated ischemic injury of aged hearts *in vivo* (**A**) The lentiviral plasmids encoding shRNAs for PHLPP-1 were injected into the left ventricular cavity of aged hearts while the aorta and pulmonary artery were cross-clamped for 50 s. Immunoblot analyses showing expressions of PHLPP-1 in young and aged hearts after 72 hours (infection with sh-PHL or sh-Con). (**B**) Young and aged hearts (infection with sh-PHL or sh-Con) were subjected to 30 minutes of *in vivo* ischemia and followed reperfusion. Representative sections of myocardial infarction and the percent of infarct size after 4 hours reperfusion. (**C**-**F**) Heart rate, left ventricular end diastolic pressure (LVEDP) and ±LVdp/dt determined by multi-channel physiological signal acquisition processing system after 2 hours reperfusion. (**G**) Cardiac function determined by echocardiography after 24 hours reperfusion. LVEF, left ventricular ejection fraction (*P < 0.05 vs. Sham; ^#^*P* < 0.05 vs. Aged I/R+Ad-sh-Con. Values are means ± S.E., n = 5 per group).

Since deletion of PHLPP-1 enhanced Akt phosphorylation, we next determined whether removing the break on Akt activation imposed by PHLPP-1 accentuates the toleration against MI/R injury in aged hearts. We knocked down PHLPP-1 *in vivo* using lentiviral system carrying a shRNA directed towards *Phlpp-1*(sh*Phlpp-1*). The lentiviral sh-PHLPP-1was delivered via intra-ventricular cavity injections while the aorta and pulmonary artery were cross-clamped for 50 s [25, 26]. After 72 h of sh-PHLPP-1 or sh-Con injection in hearts, aged rats were subjected to cardiac surgery (MI/R). The protein level of cardiac PHLPP-1 *in vivo* was decreased in sh-PHLPP-1 infected aged hearts compared with that in sh-Con injected hearts after 72 h infection (Fig.5A). Aged hearts with PHLPP-1 knockdown exhibited significantly reduced infarct size (Fig.5B). Meanwhile, PHLPP-1 knockdown *in vivo* did not affect heart rate among all groups (Fig.5C). As expect, PHLPP-1 knockdown induced comparable amelioration in cardiac LVEDP and ±LVdp/dt_max_ during reperfusion in the aged hearts (Fig.5D-F). Echocardiography demonstrated that PHLPP-1 knockdown significantly improved the LV ejection fraction after 24 hours of reperfusion in the aged hearts (Fig.5G). These data, being consistent with our *in vitro* data, confirmed that PHLPP-1-mediated inhibitory effect on Akt activation may be the cause of (at least part of) ischemic intolerance in aged hearts. Inhibition of PHLPP-1 should have beneficial effects for protecting aged heart against MI/R injury.

### Akt response to insulin was ameliorated by exercise training in aged hearts

**Figure 6:**
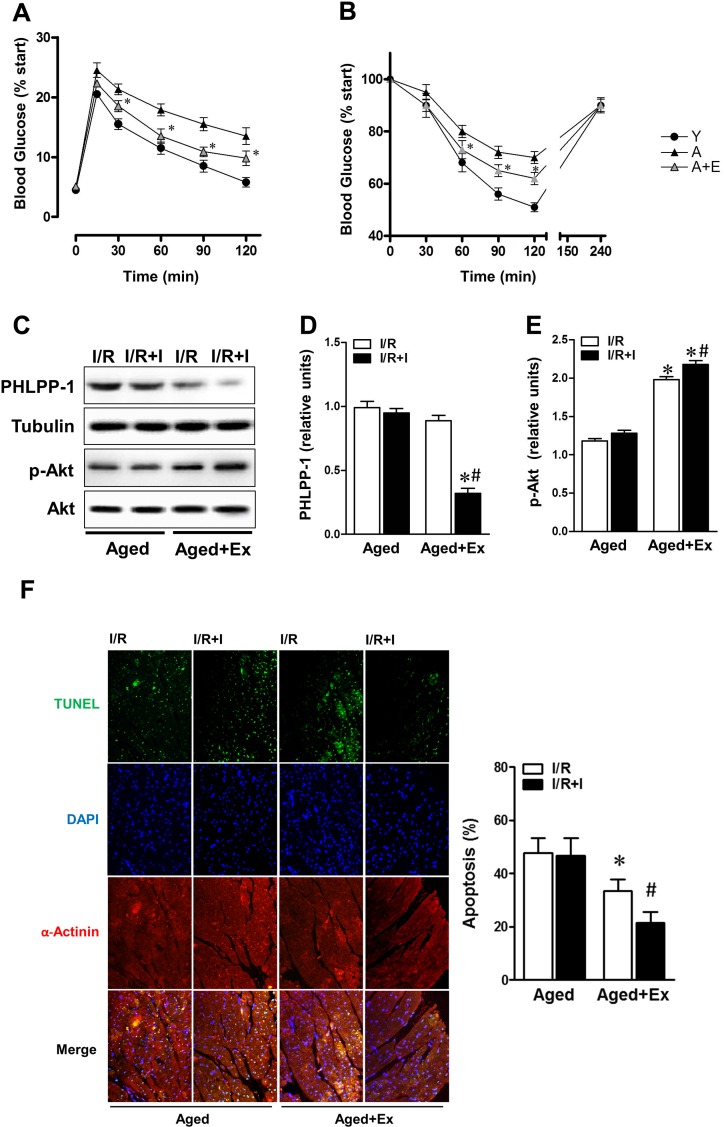
Exercise training reversed response to insulin of aged hearts Aged rats were experienced exercise training for 10 weeks. (**A**) Intraperitoneal glucose tolerance test (IPGTT) and (**B**) insulin sensitivity test (IST) determined in rats of the indicated group (*P < 0.05 vs. untrained aged group). (**C**) Trained or untrained aged hearts were subjected to *in vivo* ischemia and reperfusion. Immunoblots showing PHLPP-1 and Akt phosphorylation. (**D**-**E**) Quantification of PHLPP-1 and Akt phosphorylation immunoblots results (*P < 0.05 vs. untrained aged+ I/R+Ins; ^#^*P* < 0.05 vs. trained aged+I/R). (**F**) Cardiomyocyte apoptosis determined by TUNEL (*P < 0.05 vs. untrained aged+ I/R; ^#^*P* < 0.05 vs. trained aged+I/R, Values are means ± S.E., n = 5 per group).

Previous studies have demonstrated that shortage of protection induced by insulin was associated with weaken insulin sensitivity, and aerobic exercise partially improves insulin intolerance/sensitivity in diabetes and aging [27, 28]. Therefore we hypothesized whether we can enhance Akt activation to reverse ischemic intolerance through improving the response of aged rats to insulin. With references to former methods [29], the aged rats were experienced swimming exercise training 1 hour per day for 10 weeks in our study. IPGTT and IST data manifested that whole body insulin sensitivity of aged rats was effectively improved after long-term exercise training (Fig.6A, B). When subjected to *in vivo* MI/R, administration of insulin during reperfusion, protein level of PHLPP-1 was partially downregulated in exercise group (Fig.6C,D), in correspondence with comparable enhancement in Ser473 phosphorylation of Akt (Fig.6C, E). As a consequence, MI/R-induced cardiomyocyte apoptosis remarkably decreased in exercise group compared with that in the sedentary group (Fig.6F). We got a clue from these data that attenuated insulin-cardioprotection resulted from impaired the inhibitory effect of insulin on PHLPP-1 expression.

### Insulin modulated the stability of PHLPP-1

**Figure 7:**
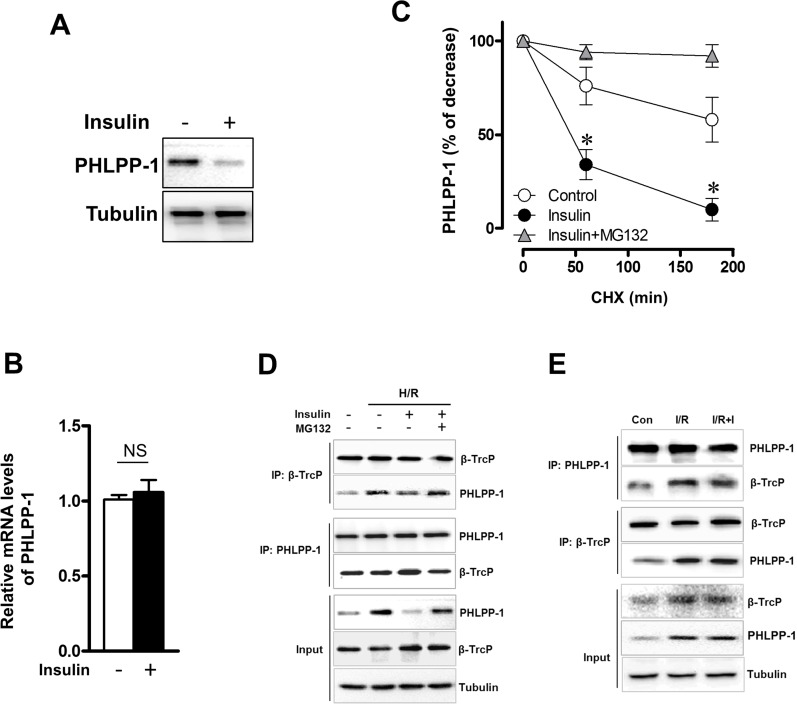
Insulin regulated the stability of PHLPP-1 in cardiomyocytes Cardiomyocytes were treated with insulin (100 nM, 3 h). (**A**) Cardiac PHLPP-1 protein level. (**B**) Quantification of PHLPP-1mRNA levels. (**C**) The quantification of PHLPP-1 abundance in cardiomyocytes with or without insulin treatment. Cardiomyocytes were treated with CHX (20 μg/mL) alone or in combination with insulin (100 nM) and MG132 (10 μM) (*P < 0.05 vs. Insulin group. Values are means ± S.E., n = 5 per group). (**D**) Cardiomyocytes were subjected to H/R with or without insulin (100 nM) and MG132 (10 μM) treatment. The interaction between PHLPP-1 and β-TrCP detected by immunoprecipitation (IP). (**E**) Aged rats were subjected to 30 minutes of ischemia and followed by 30 minutes of reperfusion with or without insulin treatment (60 U/L, intravenous infusion at 4 mL/kg per h, beginning 5 min before reperfusion). The interaction between PHLPP-1 and β-TrCP detected by immunoprecipitation (IP).

**Figure 8:**
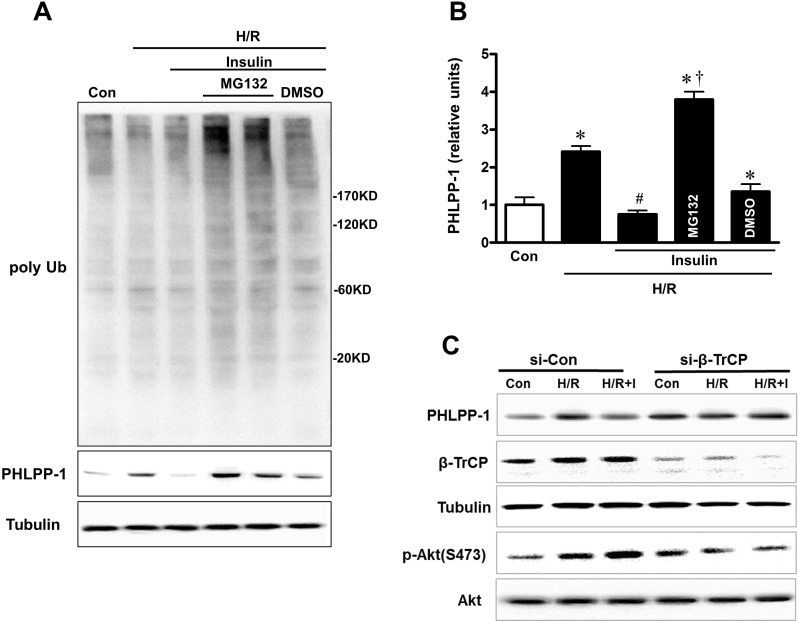
Insulin downregulated PHLPP-1 protein level through β-TrCP-mediated UPS (**A**) Representative immunoblots of ubiquitination and PHLPP-1 level with insulin and MG132 treatment. Cardiomyocytes were pre-treated with MG132, 30 min prior to H/R. (**B**) Quantification of PHLPP-1 level immunoblots results (*P < 0.05 vs. Con; ^#^*P* < 0.05 vs. H/R; ^†^*P* < 0.05 vs. H/R+Ins. Values are means ± S.E., n = 5 per group). (**C**) E3 ligase β-TrCP knockdown terminated degradation of PHLPP-1 with insulin treatment.

We have verified that PHLPP-1 protein level could be downregulated by insulin treatment in the context of MI/R. To investigate how insulin regulates PHLPP-1, we measured the protein and mRNA levels of PHLPP-1 in insulin (100 nM) -treated cultured rat ventricular myocytes (3 hr). We found that PHLPP-1 was significantly decreased at protein level but not mRNA level (Fig.7A, B). These observations led us to test whether insulin regulates PHLPP-1 at post-translational level. To test this potentiality, myocytes cells were treatment with CHX (protein synthesis inhibitor) alone or in combination with the proteasome inhibitor MG132, and the level of endogenous PHLPP-1 was monitored for 3 h with or without insulin treatment. Interestingly, insulin markedly enhanced the degradation of PHLPP-1, whereas MG132 prevented the effect of insulin on PHLPP-1(Fig.7C). These data suggested that insulin regulated the degradation of PHLPP-1 by the proteasome pathway PHLPP-1 in cardiomyocytes. It has been reported that Skp-Cullin 1-F-box protein complex (SCF) E3 ubiquitin ligase β-TrCP is responsible for the efficient degradation of PHLPP-1 [7]. Given the facts insulin down-regulated PHLPP-1 protein level, β-TrCP might play a role in insulin-induced PHLPP-1 degradation. Results from IP experiment showed that the interaction between β-TrCP and endogenous PHLPP-1 was detected in myocytes cells and insulin treatment enhanced β-TrCP interaction with PHLPP-1 resulting increased Akt phosphorylation (Fig.7D). PHLPP-1 was effectively degraded after insulin treatment during H/R, and the degradation of PHLPP-1 was blocked by MG132. In addition, in aged hearts which subjected to *in vivo* MI/R, administration of insulin during reperfusion had very little effect on β-TrCP-PHLPP-1 interaction and the degradation of PHLPP-1 was decreased (Fig.7E). These data suggested that insulin could enhance the binding of β-TrCP and PHLPP-1 to target PHLPP-1 for destruction. Immunoprecipitation and Western blotting on protein extracts from insulin-treated with or without MG132 cardiomyocytes under H/R stress, showing PHLPP-1 polyubiquitination upon UPS block (Fig.8A, B). To further explore whether β-TrCP mediate PHLPP-1 degradation in cardiomyocytes, the selective siRNA for β-TrCP was transfected into H/R-induced cardiomyocytes *in vitro*. Being consistent with MG132 treatment, transfection with siRNA of β-TrCP blocked the inhibition of insulin on PHLPP-1 during H/R (Fig.8C), suggesting that insulin downregulated PHLPP-1 protein through β-TrCP-mediated ubiquitin-proteasome system (UPS).

## DISCUSSION

We have made several observations in the present study. First, we have demonstrated that PHLPP-1 acted as an endogenous negative regulator of Akt activity and sustained presence of PHLPP-1 blocks cardioprotection by insulin-induced Akt phosphorylation during myocardial reperfusion. Second, we have provided the first evidence that insulin could enhance the binding between β-TrCP and PHLPP-1 which resulted in PHLPP-1 degradation. The decreased PHLPP-1 in turn potentiating Akt Ser473 phosphorylation may protect the hearts against MI/R injury. Thus, reciprocal inhibition of insulin and PHLPP-1 determines cardioprotective efficiency of Akt in the MI/R heart. Third, we have demonstrated that restrained effect of insulin on PHLPP-1 emerged as one of crucial causes for ischemic vulnerability in aged hearts. In contrast, pharmacological inhibition of PHLPP-1 for a relatively short time period would provide a mean of enhancing the extent or duration of Akt activation during MI/R stress through endogenous protective stimuli and this strategy should have beneficial effects in preventing cardiomyocyte loss and subsequent injury in aged hearts.

### PHLPP-1 and MI/R Injury

Akt is a cellular protective kinase in the heart. It is well established that activation of Akt protects the heart against stresses such as ischemia/reperfusion [24]. One approach to manipulating this pathway would be to increase Akt activation, while an equally feasible and potentially more selective approach would be to reduce its inactivation. Coordinate control of the balance between Akt activation and inactivation has, until recently, been poorly documented.

PHLPP-1 was discovered to be an Akt phosphatase that selectively dephosphorylates Ser473 on Akt and it was initially confirmed to regulate tumor cell survival. However, the functional significance of PHLPP-1 expression in regulating physiological and pathophysiologic responses of terminal differentiation cell (i.e. cardiomyocyte) has not been well examined [1]. In previous work, PHLPP-1 knockout mouse hearts show increased Akt and decreased infarct size following MI/R [8]. In the present study, we demonstrate that PHLPP-1 knockdown potentiates Akt phosphorylation at Ser473 and enhances Akt response to insulin, suggesting the general importance of PHLPP-1 in regulation of Akt activity in cardiomyocytes. Conversely, we also demonstrate that high-level PHLPP-1 arrests insulin-induced Akt phosphorylation in cardiomyocytes exposed to H/R. PHLPP-1 directly dephosphorylates Akt, promotes myocyte apoptosis, and aggravates the MI/R injury. These findings suggest a physiological role for this phosphatase, whereas the concomitantly diminished infarct size demonstrates its functional importance. More specifically, our present work demonstrate that increase in PHLPP-1 is responsible for weakening of insulin cardioprotection against MI/R injury. These data suggest that PHLPP-1 inhibitors could have therapeutic potential for protecting cardiomyocytes against MI/R injury. Recently, small molecule selective inhibitor against the catalytic site of PHLPP-1 have been identified [30], opening the way to strategies aimed at PHLPP targeting in therapy. By using these PHLPP-1 inhibitor, studies have shown that PHLPP-1 could become a target to restore chaperone-mediated autophagy dysfunction in aging and disease [31]. But, how can suppress PHLPP-1 *in vivo* in heart?

### Insulin and PHLPP-1 degradation

Insulin has been available for therapeutic agent in blood glucose management, and is also a key component of glucose–insulin–potassium (GIK) cocktail protecting I/R cardiomyocytes through activating PI3K-Akt signaling and recovers cardiac function, which exerts a significant endogenous protective mechanism in young adult rats [12]. Since we have realized that there existed a crosstalk between PHLPP-1 protein expression and insulin-Akt signaling whereas there is no solid evidence confirming the regulation mechanism among them, we turn attention to degradation pathway of PHLPP-1. Former study has identified that PHLPP-1 is the target of UPS-dependent protein degradation pathway, therefore, we assumed whether insulin restrain PHLPP-1 expression through acting on a sort of factors in the ubiquitination. Suppression of UPS-dependent protein degradation with protease inhibitor MG132 significantly blocked reduction of PHLPP-1 protein level with administration of insulin, which demonstrated that insulin regulated PHLPP-1 in a degradation-related manner. The UPS is a series of dynamic process regulated by three kinds of enzymes which consist of ubiquitin-activating enzyme (E1), ubiquitin-conjugating enzyme (E2), and ubiquitin ligase (E3) [32]. Given that ubiquitin ligases physically interact with target proteins providing specificity of ubiquitination [33, 34], the potential PHLPP-1-targeting ligase may be the mediator for regulation of insulin on PHLPP-1. β-TrCP, a member of F-box-containing proteins, has emerged as a key regulatory molecule with roles in cellular processes that are intimately related to tumorigenesis by regulating the proteolytic process of its substrates [34, 35]. Moreover, the research focused on cardiomyopathies has highlighted a role of β-TrCP that mediates degradation of β-catenin via ubiquitination for optimal cardiac function [36, 37]. Recent study has verified that β-TrCP ligase mediates PHLPP-1 ubiquitination and degradation controlling the level of PHLPP-1 in cancer cells [38]. Our data identified that PHLPP-1 associated with endogenous β-TrCP in cardiomyocytes, and relative more β-TrCP expressed with insulin treatment, compare to other groups. Insulin had no notably effect on expression of PHLPP-1 after loss of β-TrCP by siRNA knockdown, which provided compelling evidence that insulin is a crucial regulator of physiological inhibition of PHLPP-1 in a UPS-dependent manner via acting on β-TrCP to intensify Akt activation. The results suggest that PHLPP-1 dephosphorylates Akt to undermine cardioprotection of insulin, meanwhile, insulin suppresses PHLPP-1 to facilitate Akt activation. Thus, reciprocal inhibition of insulin and PHLPP-1 determines cardioprotective efficiency of Akt in I/R heart. Further complicating matter is the question of whether the interaction out of balance could impair the tolerance of the aged hearts to ischemic stress.

### Ischemic vulnerability of aged hearts

Aging is associated with a reduced tolerance to MI/R injury. Our data shows that aging impairs Akt activation in response to insulin administration during reperfusion. Higher myocardial PHLPP-1 expression in the aged heart as well as exacerbated myocytes death induced by H/R in the context of over-expression of PHLPP-1 *in vitro* confirmed the pivotal role of PHLPP-1 in aggravated MI/R injury of aged hearts. Then we hypothesize we can enhance myocardial Akt activity through improving insulin regulation on PHLPP-1 specifically. Clinical and animal trials have stated that moderate intensity exercise is a readily available intervention that can increase insulin action [32-35]. Moreover, Fujita S et al. have verified that aerobic exercise overcomes the age-related insulin resistance of muscle protein metabolism [35]. Similar to the former studies, we found that IPGTT and IST both confirmed that 10 weeks swimming training enhanced insulin sensitivity of aged rats. Accordingly, inhibition of insulin on PHLPP-1 protein level remarkably increased, accompanying with a little more Akt phosphorylation and much less MI/R injury following exercise training. Further, *in vivo*, PHLPP-1 knockdown contributed to improved cardiac function and decreased I/R injury in aged hearts. These findings indicate the absence of insulin inhibitory effect on PHLPP-1 in aged hearts is responsible for decreased quality for MI/R injury.

**Figure 9:**
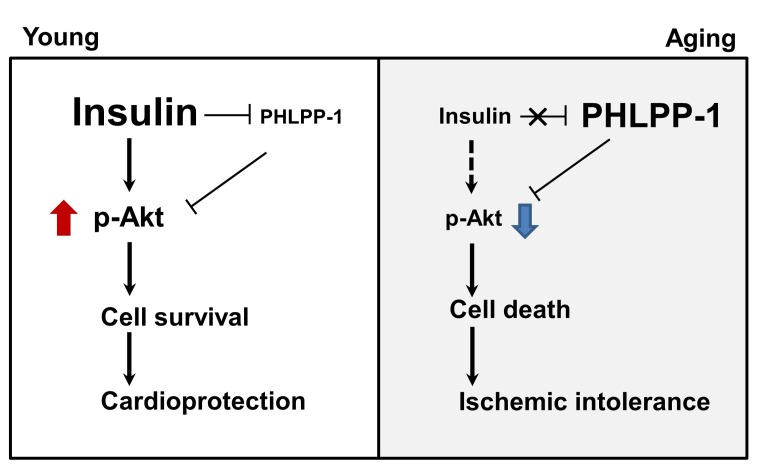
Summary of working hypothesis

In summary, PHLPP-1 dephosphorylates Akt to undermine cardioprotection of insulin. Our present study provides new insight into insulin controlling Akt activity through inhibition of PHLPP-1. Mutual inhibition of insulin and PHLPP-1 determines cardioprotective efficiency of Akt in I/R heart (Figure 9). If the interaction out of balance, aged heart possesses lower insulin effectiveness and fails to decrease PHLPP-1 protein level during MI/R, which subsequently restrains Akt activity and cardioprotection. Therefore, PHLPP-1 could be a promising therapeutic target to against ischemic vulnerability in elderly patients.

## METHODS

### Animal model of myocardial ischemia/reperfusion injury

All procedures involving experimental animals were performed following protocols approved by the Committee for Animal Research of Fourth Military Medical University and conformed to the Guide for the Care and Use of Laboratory Animals. Young (3 months) and aged (20 months) male Sprague-Dawley (SD) rats were subjected to myocardial ischemia/reperfusion (MI/R), as described previously [18]. Briefly, rats were anesthetized with pentobarbital sodium (50 mg/kg, i.p.) and ventilated on a Harvard rodent respirator via a tracheostomy. A midline sternotomy was performed, and the left anterior descending artery (LAD) was ligated with a 5-0 silk suture. Electrocardiograms confirmed ischemic repolarization changes (ST-segment elevation) during coronary occlusion. After 30 minutes of MI, the slipknot was released, and the myocardium was reperfused for 2 hours, 4 hours or 24 hours (for cardiac function, infarct size determination and echo-cardiography). Intravenous infusion of insulin was at 4 mL/kg (60 U/L) per hour beginning 5 minutes before reperfusion. Hemodynamics indexes were recorded for the entire procedure from multi-channel physiological signal acquisition processing system (PowerLab, AD Instruments) that was connected to pressure transducer.

### Determination of myocardial apoptosis and infarction size

MI size was determined by the Evans blue/TTC double staining method as described previously [10]. Hearts were fixed, sectioned, and photographed using a microscope and analyzed by planimetry with Image J. The infarct size was calculated as infarct area divided by area at risk (IF/AAR). Myocardial apoptosis was determined by terminal deoxynucleotidyl transferase–mediated dUTP nick-end labeling (TUNEL) staining using an In Situ Cell Death Detection Kit (Roche Molecular Biochemicals, USA) according to the protocol provided by the manufacturer and modified as described previously [10, 19]. Caspase-3 activities were measured using the Caspase 3/CPP32 Colorimetric Assay Kit (cat# K106-100, BioVision) according to the manufacturer's instructions.

### Swimming exercise training

Aged rats assigned to an exercise protocol were trained, free of loading, in a diameter and depth of 60×100 cm cylindrical tanks and water temperature was maintained at 33-35°C during swimming training. Rats swam for 15 minutes on the first day, and the swimming duration was then progressively increased to 60 minutes/day in 2-week period. This exercise training was maintained for 10 weeks and carried out 5 days per week. The rats performed the swimming training once a day during 8:00-11:00 a.m. The subsequent experiments performed after 24 hours after the last training [9, 36].

### Intraperitoneal glucose tolerance test (IPGTT) and insulin sensitivity test (IST)

The rats were deprived of food overnight (about 12 hours) or 6 hours and had free access to water. Glucose (50%, m/v) and insulin (Novo Nordisk, 300U: 3mL) were injected intraperitoneally with 2 g/kg and 0.5 U/kg body weight respectively. Blood samples were obtained from tail vein and glucose level was monitored at the indicated times (0, 30, 60, 90, 120 minutes) by blood glucose meter (LifeScan, USA).

### Primary culture of neonatal rat ventricular myocytes

Neonatal rat ventricular cardiomyocytes were isolated from 1-d-old Sprague-Dawley rats, as described previously [37]. The myocytes were plated at density of 3.2×10^4^/cm^2^ and maintained in Dulbecco's modified Eagle's medium (DMEM) supplemented with 10% FBS and 5-bromo-2′-deoxyuridine (BrdU, 0.1 mM) for 48 hours before other treatments.

### Hypoxia/reoxygenation

Cultured myocytes dishes were placed into a hypoxia chamber (Billups-Rothernberg Inc., Del Mar, Ca) under serum-free and no glucose culture Dulbecco's Modified Eagle Medium (DMEM). 5% CO_2_/95% N_2_ hypoxic gas was connected to the chamber and flushed the chamber for 10 minutes to completely deoxygenate it. The input/output clamps of the chamber were immediately clamped close. The chamber was put into a 37°C incubator for 3 hours. After hypoxia, the cells were subjected to reoxygenation for 3 hours in normoxia (5% CO_2_/95% air). At the onset of reoxygenation, myocytes were randomly exposed to one of the following treatments: vehicle; insulin (100 nM) [38].

### Transfection of cardiomyocytes with siRNA

PHLPP-1 siRNA duplexes (sense, CUA CCC AGU UCC AAA UUA UTT; antisense, AUA AUU UGG AAC UGG GUA GTT) and control non-specific siRNA duplexes (sense, UUC UCC GAA CGU GUC ACG UTT; antisense, ACG UGA CAC GUU CGG AGA ATT), and β-TrCP, (sense, CAA AUC UUC ACC GAA UCC CTT; antisense, UUC UCA CAG GCC AUA CAG GTT) were designed and purchased from Shanghai Genepharma Co. Ltd (China). Transient transfections using Lipofectamine RNAiMAX (Invitrogen) were performed following the manufacturer's instructions. The efficiency of gene knockdown was assessed by western blotting 48 h after siRNA transfection.

### Lentivirus-mediated delivery of shRNA

The lentiviral plasmids encoding shRNAs for PHLPP-1 were constructed in pGFP-C-shLenti vector (Catalog # TR30023) purchased from OriGene Technologies, Inc. A plasmid carrying a non-targeting sequence was used to create the control cells. For virus packaging, the control or PHLPP-specific shRNA constructs were co-transfected with Mission lentiviral packing mix (Sigma-Aldrich) [39]. Sprague–Dawley rats underwent gene transfer via aortic cross-clamping as previously described [25]. After dissection of the aorta and pulmonary artery, lentiviral shPHLPP-1 was injected into the left ventricular cavity through a 22G catheter while the aorta and pulmonary artery were cross-clamped for 50 s. In negative control animals, sh-Con lentivirus was injected into the left ventricular cavity while the aorta and pulmonary artery were cross-clamped for 50 s. After cross-clamping was released, the chest was closed. Myocardial PHLPP-1 expression was analyzed 72 hours later by western blotting.

### Adenovirus infection

The recombinant human-phlpp-1 over-expression plasmid expressing green fluorescent protein (GFP) was constructed and packaged into adenoviruses by GUANGZHOU INSIGHT BIOTECHNOLOGY Ltd (China). Adenovirus titer is about 1.3×10^10^ GFU/ml. At 24 hours after plating, cardiomyocytes were infected with Adv-phlpp-1-EGFP or negative control (Adv-001-EGFP) and incubated at 37°C in a humidified, 5% CO_2_ incubator. Subsequently, the cells were cultured in 10% FBS-DMEM medium for an additional 72 h before processing.

### Immunoprecipitation (IP)

Cell and heart protein extracts were prepared in IP lysis buffer containing 25mM Tris, 150mM NaCl, 1mM EDTA, 1% NP-40, 5% glycerol, pH 7.4, Na_3_VO_4_, 1 mM NaF, and 50 mM phenylmethylsulfonyl fluoride (PMSF). The sample was pre-clean with sepharose A/G beads (Santa Cruz) for 1 hour of incubation and centrifuge 1,000 rpm×5 minutes. The supernatant was subjected to PHLPP-1 antibody (1:200) immunoprecipitation overnight at 4°C and then the lysates-antibody complex were precipitated following 1 hour of incubation with sepharose A/G beads. The IP beads were washed twice in lysis buffer. Bound proteins were analyzed by western blotting.

### Determination of protein expression by western blotting

The ischemic areas of heart were collected after 30 minutes reperfusion and froze with liquid nitrogen for later signal determination. Myocardium tissue and cells samples were lysed with lysis buffe (Beyotime Institute of Biotechnology, Beijing, China). The lysates were centrifuged 12,000 rpm×15 minutes after homogenization; proteins were separated by electrophoresis on SDS-PAGE and then transferred to a polyvinylidene difluoride membrane (PVDF, Millipore, Billerica, MA). After blocking with 5% no-fat milk at room temperature for 1 hour, the PVDF membrane incubated overnight at 4°C with following antibodies: Akt (1:1,000, #9272, Cell Signaling), pAkt-S473 (1:1,000, #4058, Cell Signaling), pAkt-T308 (1:1,000, #9275, Cell Signaling), PHLPP-1(1:2,000, A304-029A, Bethyl Laboratories, Inc.) and tubulin (1:200, sc-398103, Santa Cruz). The membrane was then washed with PBST and incubated with the anti-rabbit or anti-mouse second antibody (1:5,000, Bei jing TDY Biotech CO., Ltd.) for 1 hour at room temperature. The blots were developed with an enhanced chemiluminescence detection kit (ECL, Millipore). The immunoblotting was visualized with ChemiDocXRS (Bio-Rad Laboratory, Hercules, CA) and analyzed with Quantity One software (Bio-Rad Laboratories, Inc.).

### Statistical analysis

Data were expressed as means ± S.E.. All of the statistical tests were performed with the GraphPad Prism software, version 5.0 (GraphPad Software, San Diego, CA). Differences were compared by ANOVA followed by Bonferroni correction for post hoc t-test, where appropriate. A P value of less than 0.05 was considered statistically significant.

## SUPPLEMENTARY DATA



## References

[1] Newton AC, Trotman LC (2014). Turning off AKT: PHLPP as a drug target. Annual review of pharmacology and toxicology.

[2] Diez C, Nestler M, Friedrich U, Vieth M, Stolte M, Hu K, Hoppe J, Simm A (2001). Down-regulation of Akt/PKB in senescent cardiac fibroblasts impairs PDGF-induced cell proliferation. Cardiovascular research.

[3] O'Neill AK, Niederst MJ, Newton AC (2013). Suppression of Survival Signalling Pathways by the Phosphatase PHLPP. FEBS J.

[4] Aviv Y, Kirshenbaum LA (2010). Novel phosphatase PHLPP-1 regulates mitochondrial Akt activity and cardiac cell survival. Circulation research.

[5] Donella-Deana A, Krinks MH, Ruzzene M, Klee C, Pinna LA (1994). Dephosphorylation of phosphopeptides by calcineurin (protein phosphatase 2B). European journal of biochemistry.

[6] Bononi A, Agnoletto C, De Marchi E, Marchi S, Patergnani S, Bonora M, Giorgi C, Missiroli S, Poletti F, Rimessi A, Pinton P (2011). Protein kinases and phosphatases in the control of cell fate. Enzyme research.

[7] Gao T, Furnari F, Newton AC (2005). PHLPP: a phosphatase that directly dephosphorylates Akt, promotes apoptosis, and suppresses tumor growth. Molecular cell.

[8] Miyamoto S, Purcell NH, Smith JM, Gao T, Whittaker R, Huang K, Castillo R, Glembotski CC, Sussman MA, Newton AC, Brown JH (2010). PHLPP-1 Negatively Regulates Akt Activity and Survival in the Heart. Circulation research.

[9] Moc C, Taylor AE, Chesini GP, Zambrano CM, Barlow MS, Zhang X, Gustafsson AB, Purcell NH (2015). Physiological activation of Akt by PHLPP1 deletion protects against pathological hypertrophy. Cardiovascular research.

[10] Ma H, Wang J, Thomas DP, Tong C, Leng L, Wang W, Merk M, Zierow S, Bernhagen J, Ren J, Bucala R, Li J (2010). Impaired macrophage migration inhibitory factor-AMP-activated protein kinase activation and ischemic recovery in the senescent heart. Circulation.

[11] Boengler K, Schulz R, Heusch G (2009). Loss of cardioprotection with ageing. Cardiovascular research.

[12] Yu Q, Gao F, Ma XL (2011). Insulin says NO to cardiovascular disease. Cardiovascular research.

[13] Yu Q, Zhou N, Nan Y, Zhang L, Li Y, Hao X, Xiong L, Lau WB, Ma XL, Wang H, Gao F (2014). Effective glycaemic control critically determines insulin cardioprotection against ischaemia/reperfusion injury in anaesthetized dogs. Cardiovascular research.

[14] Das UN (2003). Insulin: an endogenous cardioprotector. Current opinion in critical care.

[15] Chen T, Ding G, Jin Z, Wagner MB, Yuan Z (2012). Insulin ameliorates miR-1-induced injury in H9c2 cells under oxidative stress via Akt activation. Molecular and cellular biochemistry.

[16] Jonassen AK, Sack MN, Mjos OD, Yellon DM (2001). Myocardial protection by insulin at reperfusion requires early administration and is mediated via Akt and p70s6 kinase cell-survival signaling. Circulation research.

[17] Yao H, Han X, Han X (2014). The cardioprotection of the insulin-mediated PI3K/Akt/mTOR signaling pathway. American journal of cardiovascular drugs : drugs, devices, and other interventions.

[18] Li J, Zhang H, Wu F, Nan Y, Ma H, Guo W, Wang H, Ren J, Das UN, Gao F (2008). Insulin inhibits tumor necrosis factor-alpha induction in myocardial ischemia/reperfusion: role of Akt and endothelial nitric oxide synthase phosphorylation. Critical care medicine.

[19] Ma H, Zhang HF, Yu L, Zhang QJ, Li J, Huo JH, Li X, Guo WY, Wang HC, Gao F (2006). Vasculoprotective effect of insulin in the ischemic/reperfused canine heart: role of Akt-stimulated NO production. Cardiovascular research.

[20] Gao F, Gao E, Yue TL, Ohlstein EH, Lopez BL, Christopher TA, Ma XL (2002). Nitric oxide mediates the antiapoptotic effect of insulin in myocardial ischemia-reperfusion: the roles of PI3-kinase, Akt, and endothelial nitric oxide synthase phosphorylation. Circulation.

[21] Li M, Hirsch E (2015). Akt activation by PHLPP1 ablation prevents pathological hypertrophy by promoting angiogenesis. Cardiovascular research.

[22] Warfel NA, Newton AC (2012). PH domain Leucine-rich Repeat Protein Phosphatase, PHLPP: a New Player in Cell Signaling. The Journal of biological chemistry.

[23] Xu J, Qin X, Cai X, Yang L, Xing Y, Li J, Zhang L, Tang Y, Liu J, Zhang X, Gao F (2015). Mitochondrial JNK activation triggers autophagy and apoptosis and aggravates myocardial injury following ischemia/reperfusion. Biochimica et biophysica acta.

[24] Fujio Y, Nguyen T, Wencker D, Kitsis RN, Walsh K (2000). Akt promotes survival of cardiomyocytes in vitro and protects against ischemia-reperfusion injury in mouse heart. Circulation.

[25] Kho C, Lee A, Jeong D, Oh JG, Chaanine AH, Kizana E, Park WJ, Hajjar RJ (2011). SUMO1-dependent modulation of SERCA2a in heart failure. Nature.

[26] Hajjar RJ, Schmidt U, Matsui T, Guerrero JL, Lee KH, Gwathmey JK, Dec GW, Semigran MJ, Rosenzweig A (1998). Modulation of ventricular function through gene transfer in vivo. Proc Natl Acad Sci U S A.

[27] Li QX, Xiong ZY, Hu BP, Tian ZJ, Zhang HF, Gou WY, Wang HC, Gao F, Zhang QJ (2009). Aging-associated insulin resistance predisposes to hypertension and its reversal by exercise: the role of vascular vasorelaxation to insulin. Basic research in cardiology.

[28] Lee S, Park Y, Zhang C (2011). Exercise Training Prevents Coronary Endothelial Dysfunction in Type 2 Diabetic Mice. Am J Biomed Sci.

[29] Zhang QJ, Li QX, Zhang HF, Zhang KR, Guo WY, Wang HC, Zhou Z, Cheng HP, Ren J, Gao F (2007). Swim training sensitizes myocardial response to insulin: role of Akt-dependent eNOS activation. Cardiovascular research.

[30] Sierecki E, Sinko W, McCammon JA, Newton AC (2010). Discovery of small molecule inhibitors of the PH domain leucine-rich repeat protein phosphatase (PHLPP) by chemical and virtual screening. J Med Chem.

[31] Arias E, Koga H, Diaz A, Mocholi E, Patel B, Cuervo AM (2015). Lysosomal mTORC2/PHLPP1/Akt Regulate Chaperone-Mediated Autophagy. Molecular cell.

[32] Short KR, Vittone JL, Bigelow ML, Proctor DN, Rizza RA, Coenen-Schimke JM, Nair KS (2003). Impact of aerobic exercise training on age-related changes in insulin sensitivity and muscle oxidative capacity. Diabetes.

[33] Hawley JA, Lessard SJ (2008). Exercise training-induced improvements in insulin action. Acta Physiol (Oxf).

[34] Seals DR, Hagberg JM, Hurley BF, Ehsani AA, Holloszy JO (1984). Effects of endurance training on glucose tolerance and plasma lipid levels in older men and women. JAMA.

[35] Fujita S, Rasmussen BB, Cadenas JG, Drummond MJ, Glynn EL, Sattler FR, Volpi E (2007). Aerobic exercise overcomes the age-related insulin resistance of muscle protein metabolism by improving endothelial function and Akt/mammalian target of rapamycin signaling. Diabetes.

[36] Zhang KR, Liu HT, Zhang HF, Zhang QJ, Li QX, Yu QJ, Guo WY, Wang HC, Gao F (2007). Long-term aerobic exercise protects the heart against ischemia/reperfusion injury via PI3 kinase-dependent and Akt-mediated mechanism. Apoptosis.

[37] Feng Y, Liu Y, Wang D, Zhang X, Liu W, Fu F, Dong L, Zhang H, Li J, Gao F (2013). Insulin alleviates posttrauma cardiac dysfunction by inhibiting tumor necrosis factor-alpha-mediated reactive oxygen species production. Critical care medicine.

[38] Li J, Wu F, Zhang H, Fu F, Ji L, Dong L, Li Q, Liu W, Zhang Y, Lv A, Wang H, Ren J, Gao F (2009). Insulin inhibits leukocyte-endothelium adherence via an Akt-NO-dependent mechanism in myocardial ischemia/reperfusion. Journal of molecular and cellular cardiology.

[39] Liu J, Weiss HL, Rychahou P, Jackson LN, Evers BM, Gao T (2009). Loss of PHLPP expression in colon cancer: role in proliferation and tumorigenesis. Oncogene.

