# Supplementary material for: Mutual inhibition of insulin signaling and PHLPP-1 determines cardioprotective efficiency of Akt in aged heart
Source: Aging (Albany NY). 2016 Mar 27;8(5):873–87. doi: 10.18632/aging.100933 (PMC4931841; doi:10.18632/aging.100933)
Supplement: Supplementary file 1 [file aging-08-0873-s001.pdf]

## SUPPLEMENTARY DATA

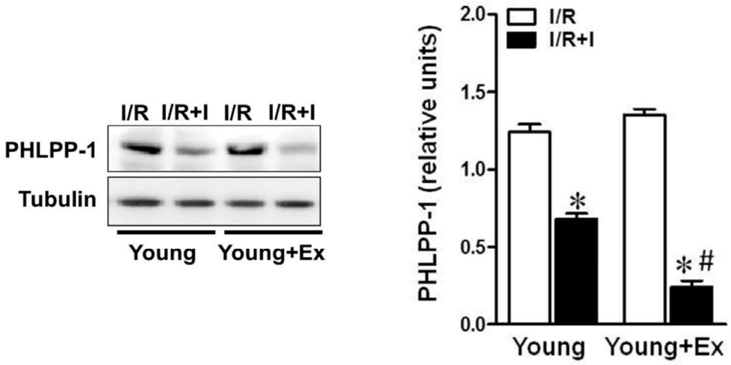


**Figure S1. Insulin downregulates PHLPP-1 level in young hearts.** Young rats were experienced exercise training for 10 weeks. Trained or untrained young hearts were subjected to *in vivo* ischemia and reperfusion. Quantification of PHLPP-1 immunoblots results shown that PHLPP-1 protein level in trained young group (young+Ex) showed no significant change comparing with untrained young group (young). But, administration of insulin during reperfusion, protein level of PHLPP-1 was markedly downregulated in untrained young group and further downregulated in exercise trained young group. Values are mean ± S.E., n=5 per group.*$P$ < 0.05 vs. Young+I/R; #$P$ < 0.05 vs. Young+Ex+I/R.
